# An organ and effective dose study of XVI and OBI cone‐beam CT systems

**DOI:** 10.1120/jacmp.v11i2.3183

**Published:** 2010-04-17

**Authors:** Daniel E. Hyer, Christopher F. Serago, Siyong Kim, Jonathan G. Li, David E. Hintenlang

**Affiliations:** ^1^ Department of Nuclear and Radiological Engineering University of Florida Gainesville FL; ^2^ Department of Radiation Oncology Mayo Clinic Jacksonville FL; ^3^ Department of Radiation Oncology University of Florida Gainesville FL USA

**Keywords:** image‐guided radiation therapy, patient dose, point radiation detector

## Abstract

The main purpose of this work was to quantify patient organ doses from the two kilovoltage cone beam computed tomography (CBCT) systems currently available on medical linear accelerators, namely the X‐ray Volumetric Imager (XVI, Elekta Oncology Systems) and the On‐Board Imager (OBI, Varian Medical Systems). Organ dose measurements were performed using a fiber‐optic coupled (FOC) dosimetry system along with an adult male anthropomorphic phantom for three different clinically relevant scan sites: head, chest, and pelvis. The FOC dosimeter was previously characterized at diagnostic energies by Hyer et al. [Med Phys 2009;36(5):1711–16] and a total uncertainty of approximately 4% was found for in‐phantom dose measurements. All scans were performed using current manufacturer‐installed clinical protocols and appropriate bow‐tie filters. A comparison of image quality between these manufacturer‐installed protocols was also performed using a Catphan 440 image quality phantom. Results indicated that for the XVI, the dose to the lens of the eye (1.07 mGy) was highest in a head scan, thyroid dose (19.24 mGy) was highest in a chest scan, and gonad dose (29 mGy) was highest in a pelvis scan. For the OBI, brain dose (3.01 mGy) was highest in a head scan, breast dose (5.34 mGy) was highest in a chest scan, and gonad dose (34.61 mGy) was highest in a pelvis scan. Image quality measurements demonstrated that the OBI provided superior image quality for all protocols, with both better spatial resolution and low‐contrast detectability. The measured organ doses were also used to calculate a reference male effective dose to allow further comparison of the two machines and imaging protocols. The head, chest, and pelvis scans yielded effective doses of 0.04, 7.15, and 3.73 mSv for the XVI, and 0.12, 1.82, and 4.34 mSv for the OBI, respectively.

PACS number: 87.57.uq

## I. INTRODUCTION

Advances in radiation treatment delivery, such as intensity‐modulated radiation therapy (IMRT), have made it possible to deliver large doses of radiation to a treatment volume with a high degree of conformity. While a highly conformal treatment offers the advantage of sparing surrounding normal tissue, this benefit can only be realized if the patient is accurately positioned during each treatment session. The need to accurately position the patient has led to the development and use of gantry mounted kilovoltage cone beam computed tomography (CBCT) systems.^(1)^ ^,^ ^(2)^

These systems are used to acquire high resolution volumetric images at the time of treatment, which can then be digitally registered with the planning CT in order to determine the appropriate shifts of the treatment table required to accurately align the patient. To date, two such systems are commercially available, the X‐ray Volumetric Imager (XVI, Elekta Oncology Systems, Crawley, UK) and the On‐Board Imager (OBI, Varian Medical Systems, Palo Alto, CA).

While CBCT is a very useful tool for ensuring that a patient is properly aligned prior to treatment, daily use in a high fraction therapy regimen will result in an associated imaging radiation dose. In an effort to quantify the dose from CBCT, several authors have performed dose measurements in cylindrical acrylic phantoms, reporting measured doses as high as 10 cGy from a single scan.^(^ ^3^ ^–^ ^7^ ^)^ While these studies provide useful dose metrics for which to compare different imaging protocols and equipment, the reported doses should be taken with great caution as humans are substantially different in size, shape, and attenuation from the acrylic phantoms used in these studies.^(8)^ To more accurately quantify patient dose, several other authors have performed dose measurements within commercially available anthropomorphic phantoms.^(^ ^9^ ^–^ ^13^ ^)^ The present study aims to build on these previous studies by performing a comprehensive set of organ dose measurements in an adult male anthropomorphic phantom for both the XVI and OBI CBCT systems using currently available manufacturer‐installed protocols and software (Version 4.0 for the XVI and Version 1.4.13.0 for the OBI). Previous studies with the XVI did not include the use of a bow‐tie filter (and associated protocols to account for the filter), and a recent software upgrade to the OBI has reduced the total tube current time product used and now offers a half rotation scan for head protocols. These technical changes to imaging parameters are intended to reduce the patient dose from cone‐beam imaging, which is of increasing clinical significance with daily image acquisitions.

Organ doses were quantified for 26 organs using an anthropomorphic phantom. Most of the 26 organs were chosen based on recommendations by the International Commission on Radiological Protection (ICRP) Publication 103 for the evaluation of effective dose.^(14)^ As is common in medical physics, the effective dose was adopted as a way to assess whole body dose for a specific patient, and to compare different imaging protocols and equipment.^(15)^ It is important to stress that because only a male phantom was used to derive the effective dose and the weighting factors defined by the ICRP are sex averaged, the reader should be cautious if using this value in risk estimation models. In addition to the dosimetry measurements, a comparison of image quality for each of the factory‐installed protocols was performed using a Catphan 440 image quality phantom.

## II. MATERIALS AND METHODS

### A. CBCT systems evaluated

This study evaluated dose delivered from both the Elekta XVI and Varian OBI CBCT systems. The two systems are similar in design with a kV X‐ray source and flat‐panel detector mounted to the gantry and sharing a common axis of rotation with the MV treatment beam. A brief description of each system is presented in the following text; however, readers looking for a more complete description are referred to Song et al.^(7)^

**Figure 1:**
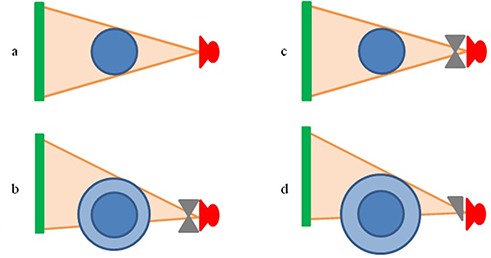
Scanning configuration for each imaging protocol used. As seen, the collimation was changed and the detector panel was shifted orthogonal to the kV beam axis when a larger FOV was required. XVI head scan, no bow‐tie filter, imaging panel centered, 27 cm FOV; (b): XVI chest and pelvis scan, bow‐tie filter, imaging panel shifted, 41 cm FOV; (c): OBI head scan, bow‐tie filter, imaging panel centered, 25 cm FOV; (d): OBI chest and pelvis scan, half bow‐tie filter, imaging panel shifted, 45 cm FOV.

The XVI system utilizes an amorphous silicon/cesium iodide (aSi/CsI) flat‐panel detector with a 1024×1024 array of 0.4 mm elements. The X‐ray source is mounted to a retractable arm with a fixed source to isocenter distance of 100 cm and offers several different collimators and kV filter combinations which are interchangeable based on the desired volume to be scanned. For the head scan, a “head and neck” protocol was selected along with an S20 collimator, which yielded an axial field‐of‐view (FOV) of 27 cm (as shown in Fig. 1(a) with a length of 26 cm along the longitudinal axis. Chest scans were performed with a “chest” protocol and an M20 collimator, which yielded an axial FOV of 41 cm with a length of 26 cm. Pelvis scans were performed with a “prostate” protocol and an M10 collimator, which yielded an axial FOV of 41 cm with a length of 12.5 cm. In addition to changing the collimators for each protocol, the chest and pelvis scans also required that the detector panel be shifted orthogonal to the kV beam axis to achieve the increased FOV^(16)^ (as shown in Fig. 1(b). Additionally, a bow‐tie filter (designation F1) was utilized for both the chest and pelvis scans.

The OBI system utilizes an amorphous silicon (aSi) flat‐panel detector with a 2048×1536 array of 0.194 mm elements. The X‐ray source for the OBI is mounted to a retractable arm with a variable source to isocenter distance of 80 to 100 cm. However, during this study, all measurements were performed with a fixed source to isocenter distance of 100 cm. The operation of the OBI unit is similar to the XVI unit, but offers only two different scan modes: full‐ and half‐fan. In the full‐fan mode, a full bow‐tie filter is used and the collimation is automatically adjusted by a set of dynamic jaws mounted to the X‐ray source. The resulting FOV is 25 cm with a length of 18 cm, as shown in Fig. 1(c). In the half‐fan mode, the detector panel is shifted orthogonal to the kV beam axis and a half bow‐tie filter is used; resulting in a FOV of 45 cm with a length of 16 cm, as shown in Fig. 1(d). The full‐fan scan was used for head imaging due to its smaller FOV, while the half‐fan scan was reserved for chest and pelvis imaging.

For each machine, three clinically relevant scan sites were investigated: head, chest, and pelvis. Technical settings for each scan remained unchanged from the manufacturer‐installed protocols currently used at our institutions. Table 1 summarizes the measured half‐value layers (HVLs) in aluminum, as well as the relevant scan parameters used for each protocol. From this data, it can be seen that the manufacturers have selected widely varying technical settings, even for the same anatomical sites.

**Table 1:** Nominal technical settings and measured HVLs for each imaging protocol investigated.

*Scan Site*	*XVI*			*OBI*		
	*Head*	*Chest*	*Pelvis*	*Head*	*Chest*	*Pelvis*
kV Collimator	S20	M20	M10	‐	‐
kV Filter^a^	F0	F1	F1	Full bow‐tie	Half bow‐tie	Half bow‐tie
kVp	100	120	120	100	110	125
mA	10	40	64	20	20	80
ms/projection	10	40	40	20	20	13
# of Projections	361	643	643	360	655	655
Total mAs	36.1	1028.8	1646.1	145	262	680
Meaured HVL (mm Al)	5.9	8.9	8.9	5.4	5.7	6.4
Acquisition Angle^b^	350°‐190°	273°‐269°	273°‐269°	88°‐292°	88°‐92°	88°‐92°
	cw	cw	cw	cw/ccw	cw/ccw	cw/ccw
Acquisition Time	~70 s	~120 s	~120 s	~30 s	~60 s	~60 s
Axial Field of View (cm)	27	41	41	25	45	45
Long. Field of View (cm)	26	26	12.5	18	16	16

a. On the XVI, F0 is the designation for no filter and F1 is the designation for a full bow‐tie filter.

b. For acquisition angle, cw means clockwise and ccw means counter‐clockwise, as viewed from the patient table.

### B. Anthropomorphic phantom

The anthropomorphic phantom used in this study was based on a hybrid computational model representing a 50th percentile adult male. In this context, hybrid refers to a cross between stylized and tomographic phantoms, exploiting the advantages of each (scalability of stylized phantoms and anatomical realism of tomographic phantoms). The hybrid model originated from a tomographic dataset of a 36‐year‐old Korean male (176 cm height, 73 kg weight), but was subsequently modified using non‐uniform rational B‐spline (NURBS) surface modeling and methods previously described by Lee et al.^(17)^ ^,^ ^(18)^ Modifications were done to match the anthropometric dimensions and organ masses as defined by the International Commission on Radiological Protection (ICRP) Report 89 reference data for a 50th percentile adult male.^(19)^

**Figure 2:**
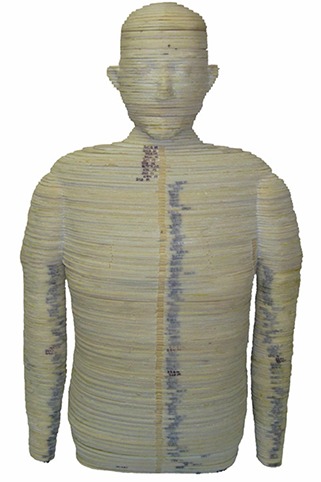
Completed torso of the 50th percentile adult male anthropomorphic phantom.

Techniques previously described by Winslow et al.^(20)^ were used to construct the physical phantom from this hybrid dataset in 5 mm thick slices using three tissue‐equivalent materials that mimic the dosimetric characteristics of a real human body. These materials include soft tissue‐equivalent substitute (STES), lung tissue‐equivalent substitute (LTES), and bone tissue‐equivalent substitute (BTES). While the hybrid dataset included over three‐hundred 5 mm thick axial slices, the lack of radiosensitive organs in the legs justified their exclusion from fabrication. For this reason, the completed phantom includes approximately 200 slices, ranging from the crown of the head to mid‐thigh. The torso of the completed phantom is shown in Fig. 2. It should be noted that while the phantom is pictured with arms attached, the arms were not used during data acquisition as patients are typically positioned such that their arms are not in the radiation field during treatment. To hold the phantom together during imaging, a patient immobilization vacuum bag was utilized.

### C. Dosimetry system

**Figure 3:**
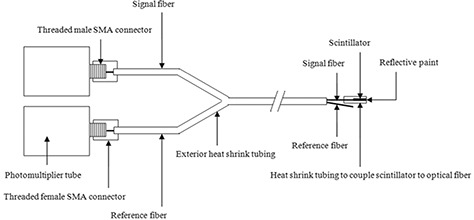
Schematic detailing the components of the dosimetry system. (The exterior heat shrink tubing extends past the scintillator to restrict any ambient light from entering the assembly, but is not shown to aid in visualization.)

A fiber‐optic coupled (FOC) dosimetry system was used for all organ dose measurements in this study. Previously described by Hyer et al.,^(21)^ the dosimeter consists of a water‐equivalent plastic scintillator (BCF‐12, Saint‐Gobain Crystals, Nemours, France), 500 μm in diameter and 2 mm in length, coupled to a 400 μm diameter optical fiber that is 2 m in length (400‐UV, Ocean Optics Inc., Dunedin, FL). The optical fiber acts as a light guide to transmit scintillation photons to a photomultiplier tube (PMT) (H7467, Hamamatsu Corporation, Bridgewater, NJ). A second optical fiber with no scintillator, referred to as the reference fiber, was also used to account for the stem effect commonly seen in FOC dosimeters as a result of the native fluorescence of the optical fiber itself.^(^ ^22^ ^–^ ^24^ ^)^ This reference fiber was integrated into the FOC dosimeter assembly using a single piece of heat shrink tubing, which was bifurcated at its end to allow each fiber to be connected with its respective PMT. By using a reference fiber, the fluorescence of the optical fiber itself can be quantified and subtracted from the FOC dosimeter signal (which includes components from both the plastic scintillator and optical fiber fluorescence), thus leaving only the signal from the scintillator. Coupling the optical fibers to the readout PMTs was achieved by installing a female SMA (SubMiniature version A) connector to each fiber and a male SMA connector on the face of each PMT. For clarification, a schematic detailing the components of the dosimetry system is shown in Fig. 3. To increase the speed of data collection, a total of two FOC dosimeters were used in this study, requiring the use of four identical PMTs. To interface with this PMT array, a custom MATLAB (MathWorks Inc., Natick, MA) computer program was also developed.

Calibration of the FOC dosimeters, in net counts per unit air kerma, was performed at different depths in water using a calibrated (traceable to an accredited dosimetry laboratory) 15 cm^3^ pancake ion chamber and associated electrometer (chamber model 96035B, electrometer model 35050A, Keithley Instruments Inc., Cleveland, OH). The calibration was performed at several depths in order to account for the energy dependence of the dosimeter, as the beam quality changes with depth due to beam hardening and the addition of a scatter component. Results indicated that the sensitivity of the dosimeter varied by approximately 5% across the depth range investigated (surface to 16 cm). Therefore, the calibration factor measured at a depth of 8 cm was chosen for all in‐phantom dose measurements, as it offered a compromise between the surface and deepest calibration factors relevant to the dimensions of the phantom and also minimized the uncertainty of the dose measurements due to the energy dependence of the dosimeter (i.e. ±2.5%). Using this calibration factor, the absorbed dose to soft tissue, $D_{abs}$, was calculated using Eq. 1:

(1) $$D_{abs}=\frac{C_{net}}{CF}\left[{\left(\frac{{\overset{ˉ}{\mu }}_{en}}{\rho }\right)}_{air}^{soft\;tissue}\right]$$

where $C_{net}$ is the net counts from the dosimeter, *CF* is the calibration factor measured at a depth of 8 cm in net counts per unit air kerma, and ${\left(\frac{{\bar{\mu }}_{\text{en}}}{\rho }\right)}_{\text{air}}^{\text{soft}\;\text{tissue}}$ is the ratio of average mass energy absorption coefficients of soft tissue to air at the measured HVL.^(25)^

The maximum uncertainty for in‐phantom FOC dosimeter measurements was estimated to be approximately 4%. Contributing factors included: (1) energy dependence of ~2.5% as the beam quality varies within the phantom due to hardening and scatter; (2) angular dependence of ~2.5% averaged over an entire revolution from a normal‐to‐axial irradiation in a scatter medium; (3) reproducibility variation of ~1% for diagnostic level exposures; and (4) variation of ~1.5% in the dosimeters response depending on the bend radius of the optical fiber.^(21)^

### D. Calculation of organ doses

To achieve clinically relevant dose values, the anthropomorphic phantom was set up and imaged in a close approximation to a real patient for the particular anatomical region being treated. For head scans, the phantom was aligned with the center of the brain at isocenter of the treatment beam. For chest scans, the isocenter was placed in the center of the body at an axial plane near the center of the lungs. For pelvis scans, the center of the prostate was placed at isocenter. To ensure reproducible results, care was always taken to accurately position the phantom on each machine before beginning data acquisition.

**Figure 4:**
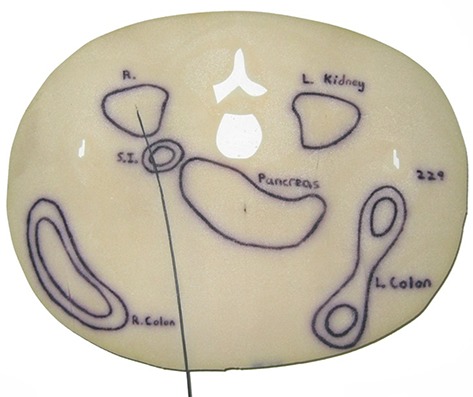
Axial slice of the physical phantom with an FOC dosimeter installed in the right kidney for dose measurements (displayed in prone position but phantom was oriented in supine position for measurements).

To aid in the positioning of dosimeters, organ locations were transferred onto the phantom using a permanent marker from full‐scale printouts of the hybrid dataset. Thin slits were cut into the axial slices of the phantom to allow placement of both the FOC dosimeters and passage of the optical fiber that connects the scintillator to the readout PMT (see Fig. 4). For small organs, absorbed dose values obtained near the centroid of the organ were adopted as the organ dose. For larger organs where dose gradients are a concern, the organ was equally subdivided and dosimeters were placed near the centroid of each of these subdivisions. The average reading from all dosimeters placed in the organ was then adopted as the average organ dose. Table 2 lists all of the organs investigated, as well as the number of measurement locations used to evaluate the dose to each organ.

**Table 2:** Organs investigated and number of measurement locations

*Tissue/Organ*	*Measurement Points*
Brain	4
Salivary glands	6
Thyroid	1
Esophagus	6
Lung	8
Breast	2
Liver	4
Stomach	4
Colon	10
Bladder	1
Gonads (testes)	2
Bone marrow	19
Bone surface	19
Skin	2
Remainder Organs:	
Extrathoracic region	4
Oral mucosa	1
Thymus	1
Heart	1
Spleen	2
Adrenals	2
Gall bladder	1
Kidneys	2
Pancreas	1
Small intestine	6
Prostate	1
Other Organs:	
Lens	2

In order to calculate the average dose to the red bone marrow, weighting factors were applied to individual red bone marrow sites (a total of 19 points in 12 sites) based on their percent weight contribution to the total red bone marrow, as shown in Eq. 2:

(2) $$D_{RBM}=\sum_{i}D_{abs,i}*A_{i}$$

where $D_{abs,i}$ is the absorbed dose to soft tissue at location *i*, and $A_{i}$ is the weight fraction of red bone marrow located at location *i*. The absorbed dose to soft tissue was used to approximate the dose to the red bone marrow because the mass energy‐absorption coefficient for red bone marrow is within 5% of the mass energy‐absorption coefficient for soft tissue at X‐rays energies greater than 30 keV.^(26)^ Dose to the bone surface was calculated in a similar fashion using Eq. 2, except $A_{i}$ was replaced with $E_{i}$ – the weight fraction of endosteum at location *i*. Table 3 shows weight fractions of red bone marrow and endosteum used for the bone sites investigated in this study, taken from the development of the hybrid computational phantom series.^(27)^

**Table 3:** Weight fractions of red bone marrow, $A_{i}$, and endosteum, $E_{i}$, for various locations of interest of the 50th percentile adult male hybrid phantom.

	$A_{i}$	$E_{i}$
Cranium	0.049	0.118
Cervical vertebrae	0.032	0.021
Humerus‐proximal	0.031	0.027
Clavicles	0.011	0.007
Scapula	0.091	0.070
Ribs	0.102	0.042
Sternum	0.026	0.010
Thoracic vertebrae	0.130	0.048
Lumbar vertebrae	0.130	0.054
Os coxae	0.265	0.173
Sacrum	0.081	0.037
Femur‐proximal	0.045	0.065

The absorbed dose to lung tissue was calculated using Eq. 3:

(3) $$D_{lung}=D_{abs}\left[{\left(\frac{{\overset{ˉ}{\mu }}_{en}}{\rho }\right)}_{soft\;tissue}^{lung}\right]$$

where ${\left(\frac{{\bar{\mu }}_{\text{en}}}{\rho }\right)}_{\text{soft tissue}}^{\text{lung}}$ is the ratio of average mass energy absorption coefficients of lung tissue to soft tissue at the measured HVL.^(25)^ The absorbed dose to skin (for the whole body) was calculated similarly using Eq. 4:

(4) $$D_{skin}=D_{abs}*\frac{A_{irradiated}}{A_{total}}*\left[{\left(\frac{{\overset{ˉ}{\mu }}_{en}}{\rho }\right)}_{soft\;tissue}^{skin}\right]$$

where ${\left(\frac{{\bar{\mu }}_{\text{en}}}{\rho }\right)}_{\text{soft tissue}}^{\text{skin}}$ is the ratio of average mass energy absorption coefficients of skin to soft tissue at the measured HVL^(25)^ and $\frac{A_{\text{irradiated}}}{A_{\text{total}}}$ is the ratio of irradiated skin area to the gross surface area of the entire phantom. The gross surface area of the phantom used in this study was estimated to be 1.83 m^2^, using the computational model as a reference. The irradiated area was also estimated using the computational model as a reference and values of 0.12 m^2^, 0.32 m^2^, and 0.20 m^2^ were used for the head, chest, and pelvis scans, respectively. The absorbed dose at the surface of the phantom was evaluated by placing two dosimeters in the primary field at the anterior and left lateral sides of the phantom and averaging the readings from these two dosimeters.

### E. Calculation of effective dose

The measured organ doses along with weighting factors from the ICRP Publication 103 were used to calculate a reference male effective dose for each protocol. Equation 5 shows the formula recommended for evaluating the effective dose:

(5) $$E=\sum_{T}w_{T}H_{T}$$

where $w_{T}$ is the tissue weighting factor and $H_{T}$ is the equivalent dose for each organ. In this case, the equivalent dose is simply the organ dose multiplied by a radiation weighting factor of unity for X‐rays. As previously mentioned, only a male phantom was used to derive the effective dose and the weighting factors defined by the ICRP are sex averaged. Therefore, this value is not intended for use in risk estimation models based on effective dose but rather for comparing different imaging protocols and equipment presented in this study.

Similar to other authors conducting effective dose calculations from organ dose measurements within anthropomorphic phantoms, the doses to two remainder organs were not measured: lymphatic nodes and muscle.^(26)^ ^,^ ^(28)^ This was due to the fact that these organs are distributed throughout the body, making it technically difficult to obtain an average organ dose measurement. Additionally, both of these organs are included in the category of “remainder organs” along with 11 other organs and, as a result, do not contribute greatly to the overall effective dose. Therefore, the weighting factor for remainder organs was simply applied to the average dose to the other 11 remainder organs.

### F. Image quality

It should be emphasized that the doses reported in this study result from using technical imaging settings provided by the manufacturer as their installed default protocols. No attempt was made to compare doses at the same image quality, but rather at clinically relevant scan settings. Consequently, there may be differences between the image quality achieved by the different systems, which was also evaluated. A Catphan 440 (The Phantom Laboratory, Salem, NY) image quality phantom was used to assess the image quality of each CBCT system as it is used clinically. Image quality was assessed using the following Catphan modules: CTP401 slice geometry and sensitometry module, and CTP592 low‐ and high‐contrast resolution module. The CTP592 module has a series of high‐resolution test patterns, ranging from 5 through 15 lp/cm, which were used for visual evaluation of spatial resolution. This module also includes a series of 0.5% low‐contrast targets for evaluating low‐contrast detectability. Unfortunately, the 0.5% low‐contrast targets were designed to evaluate fan‐beam CT systems and are too demanding for CBCT image quality tests. As a result, they cannot be seen in the reconstructed CBCT images. For this reason, the CTP401 module was used as a substitute to compare the low‐contrast detectability of each CBCT system. This module includes five acrylic spheres of varying diameters (2, 4, 6, 8, and 10 mm diameter) that have approximately 3% contrast^(29)^ and can be seen in the reconstructed images. By visually evaluating the smallest sphere that could be seen in the reconstructed images, the CTP401 module was effectively used to illustrate the differences in low‐contrast detectability between the two systems. The Catphan phantom was scanned using the same clinical protocols used during organ dose measurements. All images were reconstructed at a 5 mm slice width, with a matrix size of 512×512 pixels.

## III. RESULTS

The measured organ and effective doses from the XVI and OBI are shown in Tables 4 and 5, respectively. The “whole body” bone marrow and bone surface doses were calculated as previously discussed, while the “irradiated site” bone marrow and bone surface doses are an average of only the bone sites in the primary field. For head scans, this includes the cranium and cervical vertebrae; for chest scans, the proximal humerus, clavicles, scapula, ribs, sternum, and thoracic vertebrae; and for pelvis scans, the os coxae, sacrum, and proximal femur. The “irradiated site” skin dose is an average of the dose measured from the two dosimeters placed in the primary field on the anterior and left lateral surfaces of the phantom.

**Table 4:** Organ and effective doses from the Elekta XVI CBCT system.

*Tissue/Organ*	*Measured Organ Doses (mGy)*		
	*Head Scan*	*Chest Scan*	*Pelvis Scan*
Brain	0.70	0.49	‐
Salivary glands	0.78	1.86	‐
Thyroid	0.05	19.24	‐
Esophagus	0.02	13.56	‐
Lung	0.01	14.29	0.02
Breast	‐	16.80	‐
Liver	‐	6.58	0.19
Stomach	‐	4.68	0.23
Colon	‐	0.40	2.04
Bladder	‐	0.03	15.67
Gonads (testes)	‐	‐	29.00
Bone marrow (whole body)	0.07	5.14	1.05
Bone marrow (irradiated site)	0.80	12.42	5.50
Bone surface (whole body)	0.11	2.59	1.17
Bone surface (irradiated site)	0.80	12.42	5.50
Skin (whole body)	0.09	2.62	3.07
Skin (irradiated site)	1.34	14.92	27.88
Remainder Organs:			
Extrathoracic region	0.60	5.21	‐
Oral mucosa	0.69	1.34	‐
Thymus	0.01	14.29	‐
Heart	‐	13.87	0.10
Spleen	‐	7.17	0.20
Adrenals	‐	3.76	0.23
Gall bladder	‐	1.83	0.28
Kidneys	‐	1.20	0.31
Pancreas	‐	1.21	0.33
Small intestine	‐	0.28	1.06
Prostate	‐	‐	27.63
Other Organs:			
Lens	1.07	0.52	‐
Effective dose (msV)	0.04	7.15	3.73

**Table 5:** Organ and effective doses from the Varian OBI CBCT system.

*Tissue/Organ*	*Measured Organ Doses (mGy)*		
	*Head Scan*	*Chest Scan*	*Pelvis Scan*
Brain	3.01	0.14	‐
Salivary glands	2.42	0.30	‐
Thyroid	‐	2.38	‐
Esophagus	0.01	3.23	‐
Lung	‐	4.31	0.01
Breast	‐	5.34	‐
Liver	‐	0.97	0.28
Stomach	‐	0.74	0.30
Colon	‐	‐	3.26
Bladder	‐	‐	15.30
Gonads (testes)	‐	‐	34.61
Bone marrow (whole body)	0.28	1.29	1.14
Bone marrow (irradiated site)	3.45	3.27	5.77
Bone surface (whole body)	0.47	0.63	1.14
Bone surface (irradiated site)	3.45	3.27	5.77
Skin (whole body)	0.16	1.03	3.05
Skin (irradiated site)	2.39	5.85	27.77
Remainder Organs:			
Extrathoracic region	1.06	0.85	‐
Oral mucosa	1.39	0.38	‐
Thymus	‐	4.83	‐
Heart	‐	4.50	0.08
Spleen	‐	0.93	0.28
Adrenals	‐	0.65	0.34
Gall bladder	‐	0.14	0.52
Kidneys	‐	0.08	0.59
Pancreas	‐	0.06	0.52
Small intestine	‐	‐	1.72
Prostate	‐	‐	27.25
Other Organs:			
Lens	0.59	0.15	‐
Effective dose (msV)	0.12	1.82	4.34

The results of the image quality tests are shown in Table 6. Resolution was evaluated by the smallest resolution test pattern that could be visualized, and low‐contrast detectability was evaluated by the smallest acrylic sphere that could be visualized in the image quality phantom.

**Table 6:** Results of image quality tests for manufacturer installed protocols. Resolution was evaluated by the smallest pattern that could be visually resolved in the CTP592 module, and detectability was evaluated by the smallest acrylic sphere that could be visually resolved in the CTP401 module.

	*Head*		*Chest*		*Pelvis*	
	*XVI*	*OBI*	*XVI*	*OBI*	*XVI*	*OBI*
Resolution	>5 lp/cm	8 lp/cm	>5 lp/cm	5 lp/cm	>5 lp/cm	5 lp/cm
Detectability	>10 mm	10 mm	6 mm	4 mm	6 mm	4 mm

### A. Head

**Figure 5:**
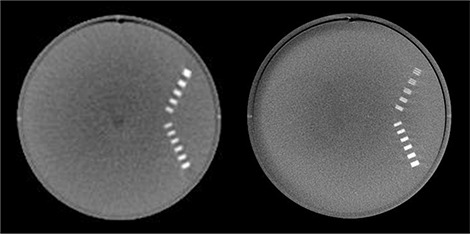
Example of reconstructed images of CTP592 resolution phantom: XVI (left), OBI (right).

The head scan covered the brain, salivary glands, extrathoracic region, oral mucosa, and parts of the esophagus. Of all the organs, the lens of the eye received the maximum dose of 1.07 mGy for the XVI, while the brain received the maximum dose of 3.01 mGy for the OBI. Image quality was assessed through visual evaluation of the reconstructed images of the Catphan phantom. The CTP592 resolution module was used to evaluate the spatial resolution. The 8 lp/cm pattern could be visually resolved in the OBI images, while none of the test patterns could be resolved in the XVI images, the largest of which was 5 lp/cm. The CTP401 module was used to evaluate low‐contrast detectability, with only the largest of the acrylic spheres (10 mm diameter) being visible on the OBI images; none of the spheres were visible on the XVI images. The images from the OBI were also noticeably sharper than those from the XVI. This can be seen in Fig. 5, which shows an example of the reconstructed images of the CTP592 resolution phantom using the head protocol for each system.

### B. Chest

The chest scan covered the lungs, breast, heart, thymus, thyroid, and parts of the esophagus, liver, stomach and spleen. The thyroid received the maximum dose of 19.24 mGy for the XVI, while the breast received the maximum dose of 5.34 mGy for the OBI. Again, the OBI showed superior spatial resolution, with the 5 lp/cm pattern visible in the reconstructed images, while none of the resolution test patterns were visible on the XVI images. The OBI also showed better low‐contrast detectability with the 4 mm diameter acrylic sphere being visible on the OBI images; the 6 mm diameter acrylic sphere was the smallest that could be seen on the XVI images. The reconstructed OBI images again appeared sharper overall than the XVI images.

### C. Pelvis

The pelvis scan covered the prostate, bladder, testes, and parts of the small intestine and colon. The testes received the maximum dose for both systems at 29 and 34.61 mGy for the XVI and OBI, respectively. The images for the pelvis protocol appeared very similar to those seen for the chest protocol, yielding similar results for resolution and low‐contrast detectability. The 5 lp/cm pattern could be resolved on the OBI images, while none of the test patterns could be resolved on the XVI images. For low‐contrast detectability, the 4 mm diameter acrylic sphere was visible on the OBI images, while the 6 mm diameter acrylic sphere was the smallest that could be seen on the XVI images. The reconstructed OBI images again appeared sharper overall than the XVI images.

## IV. DISCUSSION

It is important to differentiate the data presented in the current study with that which can be found in current literature. Kan et al.^(13)^ published an organ and effective dose study performed on the OBI using a female anthropomorphic phantom and TLDs. While the study provides useful dose information for the OBI, the recent release of OBI software version 1.4.13.0 includes new protocols which have not yet been investigated in the literature. Previous protocols for the OBI were all at a fixed tube voltage (125 kVp) and consisted of full rotation scans. The updated OBI protocols utilize body region specific tube voltages, with lower energies for less attenuating body regions. Tube voltages include 100 kV, 110 kV, and 125 kV for the head, chest, and pelvis, respectively. Additionally, the new head protocol for the OBI utilizes only a partial rotation rather than a full rotation for image acquisition. Lastly, the tube current time product for all OBI protocols has been reduced substantially. All of these changes result in lower patient doses, with the dose to many organs being reduced by 50% or more from previously published works utilizing earlier versions of the manufacturer installed protocols.^(13)^ For the XVI system, there have been very few organ dose studies published in the literature.^(4)^ ^,^ ^(11)^ Additionally, these studies report the dose to only a limited number of organs, and they were performed before the release of software version 4.0, which included the introduction of new protocols and the use of bowtie filters. While the tube current time product has been increased with the use of the new protocols to account for the added filtration, results indicate that individual organ doses for the XVI have remained comparable to the limited studies in the literature..

An interesting similarity between the XVI and OBI is the fact that they both use partial rotation scans for their head protocols (200° and 204°, respectively). On the XVI, image acquisition begins at the anterior surface of the patient and rotates around the left lateral side of the head, finishing posteriorly (when the patient is placed in a supine position). On the OBI, image acquisition moves from left to right lateral (or vice versa) while rotating around the posterior surface of the patient. Due to the choice of acquisition angles, superficial organs located on the anterior surface of the head are directly irradiated during image acquisition with the XVI but not with the OBI, resulting in comparatively higher doses to some organs for the XVI. For example, the dose to the lens of the eye on the XVI system was 52% higher than the dose to a centrally‐located organ such as the brain, while on the OBI system the dose to the lens of the eye was approximately a factor of 5 less than the dose to the brain. This result suggests modifying the manufacturer installed head protocol on the XVI such that the X‐ray tube rotates around the posterior side of the head where there are no critical organs rather than the anterior side of the head which has several superficial organs of interest.

The chest protocol for each system utilized a full rotation scan. However, the beam width for the XVI was larger than the OBI (26 cm compared to 16 cm) resulting in higher doses to organs outside of the treatment volume. The thyroid was one such organ, which is included in the larger field of view and subsequently received the highest dose of any organ during a chest scan from the XVI, but was outside of the primary field on the OBI. Contributing to the thyroid dose on the XVI was also the fact that the outer body contour was smaller in this region, resulting in less attenuation of the primary beam as the tube rotated around the body. This finding suggests reducing the beam width of the XVI in future versions if the necessary information can be gained from a narrower beam width such as is used on the OBI to avoid unnecessarily irradiating organs outside of the treatment volume. As expected, the breast received the highest dose on the OBI due to its anatomical location at the periphery of the body.

The pelvis scans for each system also utilized a full rotation. Interestingly, the OBI had organ doses similar to those measured using the XVI, even though the tube current time product was approximately a factor of 2.4 smaller. This can be attributed to the fact that the OBI has a larger beam width (16 cm compared to 12.5), resulting in additional scattered radiation, as well as a lower HVL (see HVLs in Table 1). As reported by Song et al.,^(7)^ the lower HVL of the OBI results in more dose being deposited per unit mAs than the XVI. Additionally, the testes were completely in the primary beam of the OBI, but were only partially covered by the XVI beam because of its smaller beam width, resulting in a higher gonad dose for the OBI than XVI. Similar to the chest scan, the larger beam width resulted in more organs outside of the treatment volume being irradiated and an increased scatter dose inside of the scan volume.

The effective dose for each scan is also shown at the bottom of Tables 4 and 5. The head scans for both the XVI and OBI had very low effective doses (0.04 and 0.12 mSv, respectively) due to the fact that the most of the organs in the primary field for a head scan have low weighting factors. The chest scan yielded effective doses of 7.15 and 1.82 mSv for the XVI and OBI, respectively. The higher effective dose for the XVI chest scan was due to its larger beam width, which covered more organs, and higher tube current time product, which resulted in higher overall organ doses. The pelvis scan for each system had similar effective doses at 3.73 and 4.34 mSv for the XVI and OBI, respectively. As is evident from the data presented in this study, the effective dose for each protocol was small due to the limited number of organs involved in each scan. This is especially true for the pelvis scans, where both the gonads and prostate received doses greater than 25 mGy, but most other organs received little if any dose. The effective dose was on the order of only 3–4 mSv. Therefore, organ doses are likely more meaningful for predicting future risk, such as organ specific disease, but the effective dose offers a single number to compare the different protocols and equipment quickly and easily.

The image analysis performed in this study quantified the observed image quality for each system and imaging protocol – supplementing the extensive dosimetry measurements performed. Again, it is important to remember that the protocols selected for this study were those recommended by the manufacturer for clinical use. When first comparing the reconstructed OBI images to the XVI images, it was readily apparent that the XVI images were not as sharp and had more artifacts (streaking and rings). As mentioned in the results, the OBI outperformed the XVI in both observed resolution and low‐contrast detectability for all three protocols investigated, but also delivered higher doses for two of the three protocols. However, this increased image quality did not always come at the cost of higher dose, with the chest protocol for the OBI actually yielding lower dose and better image quality.

Data presented in this study shows that daily use of CBCT for patient positioning will deliver a substantial imaging dose to organs in the primary imaging field. It should be noted that when used for daily position verification, the organ doses listed in Tables 4 and 5 must be multiplied by the total number of fractions, which can be as high as 30–40. This results in organ doses exceeding 1 Gy in some cases, such as the testes, indicating that the dose from daily CBCT imaging in a high fraction therapy regimen should be taken into consideration during the treatment planning process.

## V. CONCLUSIONS

A comprehensive set of organ dose measurements were performed using an anthropomorphic phantom and fiber‐optic coupled dosimetry system for the two commercially available kilovoltage CBCT systems (Elekta XVI and Varian OBI). The systems were evaluated by performing organ dose and image quality measurements for three clinically relevant scan sites (head, chest, and pelvis) using the latest manufacturer installed clinical protocols. Organ dose measurements demonstrated that the XVI yielded higher doses for a chest scan, while the OBI yielded higher doses for both head and pelvis scans. The dosimetric differences between these two CBCT systems are magnified over the course of a fractionated treatment with daily imaging. Specifically, the XVI chest scan delivers a dose 30 cGy higher to the lung than the OBI chest scan during a 30‐fraction regimen. For head and pelvis scans, the OBI delivers a dose 6.9 cGy higher to the brain and 16.8 cGy higher to the gonads, respectively, over the course of a 30‐fraction regimen. Image quality measurements demonstrated that the OBI provided superior image quality, with both better spatial resolution and low‐contrast detectability when using default clinical protocols. Results also showed a decrease in organ dose for the OBI when compared to previous studies which used an earlier version of the manufacturer installed clinical protocols. The new XVI protocols yielded doses similar to previously published work, despite an increase in tube current time product and the use of bow‐tie filters. In summary, the organ doses reported in this study provide practitioners a useful measure of absorbed dose from the latest manufacturer‐installed CBCT imaging protocols to weigh the added benefit of improved patient positioning against the additional radiation dose of using CBCT imaging.

## ACKNOWLEDGEMENTS

This work was supported by the U.S. Department of Energy (project award #DE‐GF07‐05ID14700) and the Center for Disease Control / TKC Integration Services (CDC Task 81, TKC 30‐07 185‐01).
